# Associations between carotid artery intima-media thickness, traditional risk factors and proteins

**DOI:** 10.1038/s44325-025-00073-7

**Published:** 2025-07-02

**Authors:** Lars Lind, Rui Zheng

**Affiliations:** Department of Medical Sciences, Uppsala University, Uppsala, Sweden

**Keywords:** Cardiovascular biology, Interventional cardiology

## Abstract

We aimed to assess the role of traditional cardiovascular risk factors on carotid artery intima-media thickness (IMT) and proteins associated with IMT. IMT was measured in 50,704 participants from the UK Biobank. Plasma levels of 2923 proteins were analyzed in 6328 individuals. Mendelian randomization (MR) analyses used genetic data on IMT, proteins, and risk factors. Observational analyses showed positive associations of LDL-cholesterol, body mass index (BMI), diabetes, and systolic blood pressure (SBP) with IMT, while HDL-cholesterol was inversely related. MR analysis confirmed causality for LDL, BMI, diabetes, but not HDL. Of 63 proteins observationally linked to IMT, 17 were replicated externally. Five proteins (BCAM, NTproBNP/NPPB, RABEPK, ACAN, FN1) were associated with IMT in MR, with RABEPK showing concordant directional relationships. MR supports causal links between LDL, BMI, diabetes, SBP, and IMT. RABEPK emerged as a potential therapeutic target, warranting further investigation.

## Introduction

Measurements of intima-media thickness (IMT) with ultrasound is commonly used in epidemiological studies to evaluate the atherosclerotic burden. Although the use of IMT as a substitute for plaque measurements has been questioned^1,2^, IMT measurements have been linked to overt atherosclerotic diseases, like myocardial infarction and ischemic stroke in epidemiological studies^3–5^. Furthermore, Mendelian randomization (MR) and colocalization studies have suggested that IMT is causally related to both myocardial infarction and ischemic stroke^6^, indicating that IMT indeed is a vascular characteristic of interest.

Risk factors for a thickened IMT is mainly the same risk factors as being well-known for myocardial infarction and ischemic stroke, such as high levels of BMI, blood pressure, LDL-cholesterol, diabetes, smoking and low levels of HDL-cholesterol^7^. However, while MR studies have confirmed that BMI, blood pressure, LDL-cholesterol, and diabetes likely are causally related to myocardial infarction and ischemic stroke^8–16^, the causal role of HDL-cholesterol as a protective factor has been questioned^11,12^. Previous MR studies have suggested causal associations of some lipids traits and BMI with IMT^17–21^, and it is of value to conduct a larger study including more cardiometabolic traits simultaneously using the UK Biobank data.

In the recent years, techniques for multiplex measurements of proteins have emerged and the circulating protein profile of many traits have been presented to deepen our understanding of disease pathophysiology and improve disease prediction^22–24^. In our previous study, we have identified eleven out of eighty-six proteins associated with thickened IMT using observational design^25^. But since that study the number of measurable proteins has grown considerably, we are now motivated to include a much greater number of proteins to be associated with IMT, using both observational and genetic analyses.

The aims of the present study were three-fold. First, to compare measured levels of traditional risk factors for IMT with genetically determined levels to evaluate causality. Second, to disclose the protein profile of a thick IMT. Third, to use MR and colocalization analyses to evaluate if some of protein-IMT associations were causal and therefore a suitable target for drug development regarding vascular diseases. For these aims, we used the UK Biobank as the major resource where almost 3000 proteins have been measured along with IMT and traditional risk factors.

## Results

**Fig. 1:**
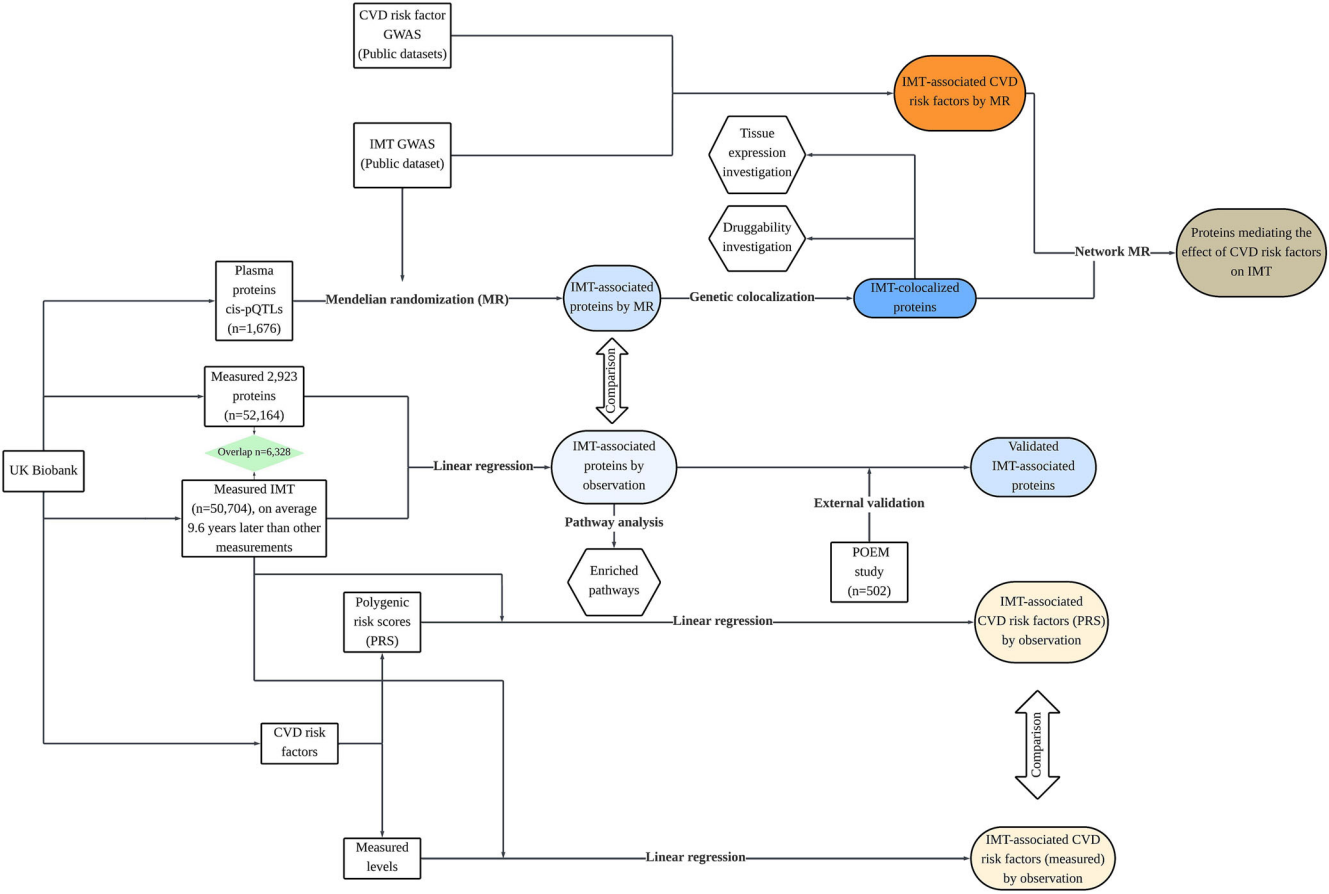
Workflow of data collection and analyses in this study. Datasets and variables used for analysis were detailed in this figure.

A flowchart describing the analyses conducted in this work can be found in Fig. 1. Basic characteristics of the studied sample is given in Table 1. For MR, 1676 cis-pQTL SNPs were included as genetic instruments (Supplementary Table 1).

**Table 1:** Basic characteristics of the UK biobank sample used in the observational analysis

Variable	Mean (SD) or proportions
Age	57.1 (8.1)
Female sex (%)	50
Townsend deprivation index	−1.3 (3.1)
Smoking (%)	Never: 54.9
	Previous: 34.5
	Current: 10.6
Race (%)	White: 94.6
	Black: 1.8
	Asian: 2.5
	Other: 1.1
Systolic blood pressure (mmHg)	139.7 (19.7)
HbA1c (mmol/mol)	36.1 (6.8)
LDL-cholesterol (mmol/l)	3.6 (.9)
HDL-cholesterol (mmol/l)	1.4 (.4)
BMI (kg/m^2^)	27.4 (4.8)
Estimated glomerular filtration rate (eGFR) (ml/min/BSA)	83.6 (15.4)
IMT (mm)	0.92 (0.21)

### Comparing the associations of measured and genetically determined levels of traditional risk factors with IMT

**Fig. 2:**
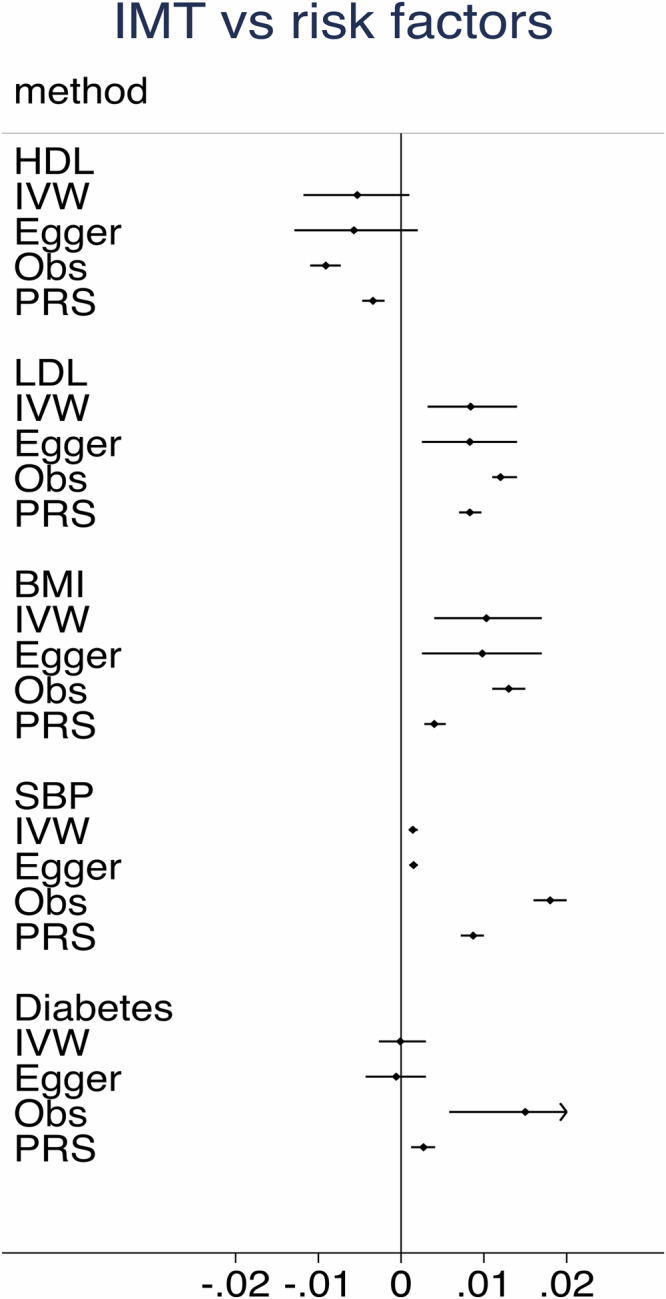
Intima-media thickness (IMT) and CVD risk factors. Relationships between IMT of the carotid artery and measured levels (Obs) or genetically determined levels of the traditional CVD risk factors, including using polygenetic risk scores (PRS), and Mendelian randomization [inverse-variance weighted (IVW) and MR Egger (Egger)] techniques. The regression coefficient (beta) and 95%CI are shown.

As could be seen in Fig. 2 and Supplementary Table 2, the observational data using measured levels of the risk factors disclosed the expected results, namely that LDL-cholesterol, BMI, diabetes, SBP were positively related to IMT, while HDL-cholesterol was related to IMT in an inverse fashion. The same pattern was also seen using the PRS for the risk factors, but in this case the estimates were generally lower than those of the measured levels. MR analyses showed LDL-cholesterol, BMI, and SBP were significantly associated with IMT in the expected fashion, while diabetes and HDL-cholesterol were not significant. IVW and MR Egger showed generally very similar estimates.

### Measured protein levels vs IMT

**Fig. 3:**
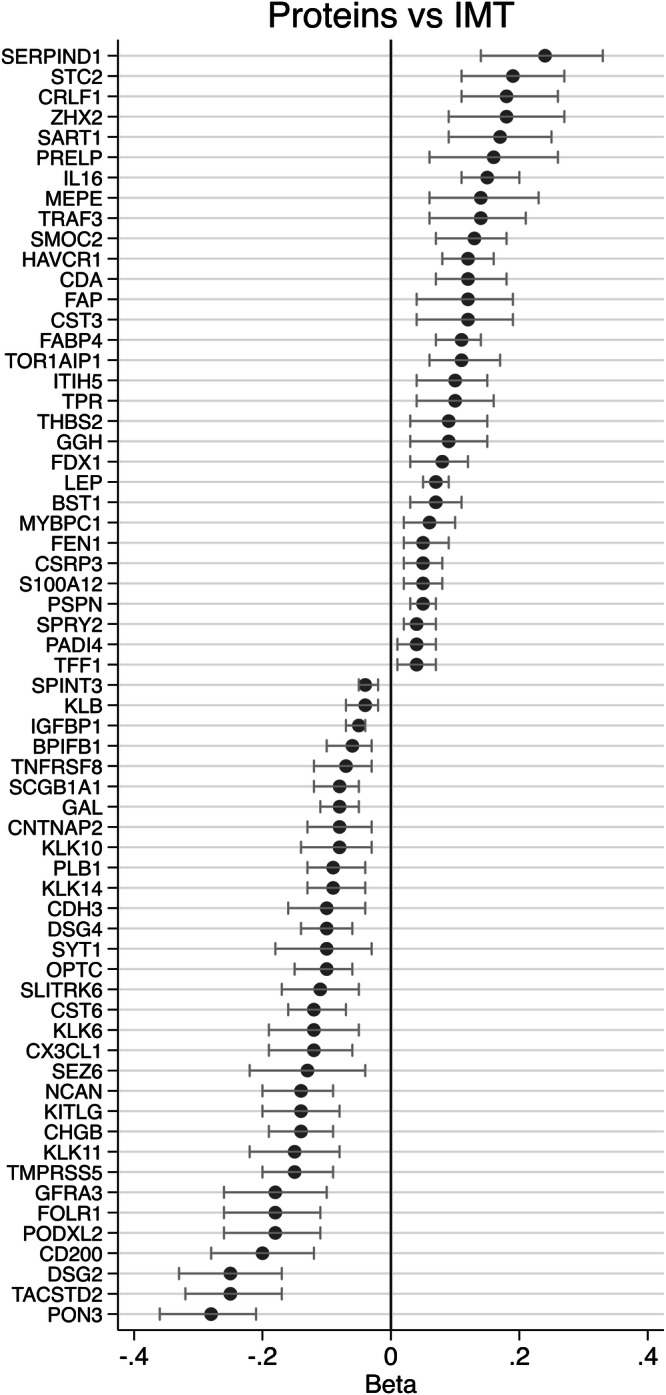
Intima-media thickness (IMT) and measured proteins. Relationships between measured protein levels and IMT. Only the 63 proteins with FDR < 0.05 in the age and sex-adjusted model and *p* < 0.05 in the multiple CVD risk factors-adjusted models are given in the figure. The estimates shown here are age, sex and fasting time-adjusted.

Of the 2923 proteins evaluated in the observational part, 478 showed FDR < 0.05 in the age and sex-adjusted model. Of those, only 63 showed *p* < 0.05 in the model also adjusted for CVD risk factors (Fig. 3 and Supplementary Table 3). Top findings were PON3, IGFBP1, HAVCR1, TACSTD2 and LEP. LEP and FABP4 did however change the sign of the estimate from positive to negative following the additional adjustment for CVD risk factors (including BMI).

When the 63 proteins of interest were subjected to pathway enrichment analysis (www.reactome.org), a number of pathways were enriched (*p* < 0.05), including regulation of insulin-growth factor transport, post-translational protein phosphorylation, white adipocyte differentiation, ATF4 activates genes in response to endoplasmatic reticulum stress, and negative regulation of FGFR4 signaling (Supplementary Table 4). Out of the 63 proteins of interest, 47 were measured in the POEM study (Supplementary Table 5), of which, 17 were found significantly (*p* < 0.05) associated with IMT, showing concordant signs of the beta coefficients with those in UKB. Top findings included FAP (Prolyl endopeptidase FAP), NCAN (Neurocan core protein), CHGB (Secretogranin-1), PON3 and GAL (Galanin peptides).

### Genetically determined protein levels vs IMT or risk factors

**Fig. 4:**
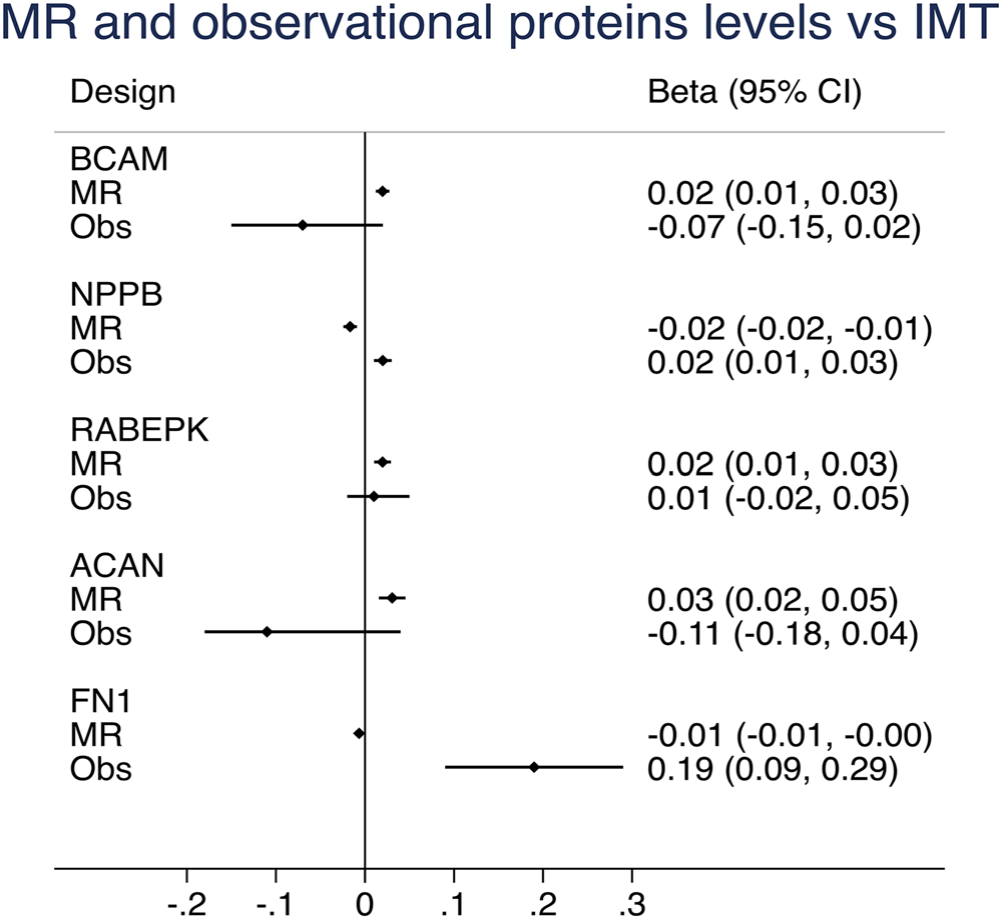
Intima-media thickness (IMT) and proteins of interest by Mendelian randomization (MR). Relationships between the five proteins being significant in the MR analysis vs IMT and the corresponding results for these proteins in the observational analysis (Obs) adjusted for age and sex.

Of the 1676 proteins evaluated in the MR analysis, only 5 showed FDR < 0.05 vs IMT (BCAM, NTproBNP/NPPB, RABEPK, ACAN, FN1)(Supplementary Table 6). None of these proteins were amongst the 63 proteins identified to be significant in the observational part (FDR-adjusted), and only RABEPK showed the same sign of direction in the MR analysis as in the observational analysis (age and sex adjusted model), as could be seen in Fig. 4. The MR sensitivity analyses showed very similar estimates of MR-Wald ratio and other MR methods using multiple SNPs. No horizontal pleiotropy was observed for all associations. For BCAM-IMT association, heterogeneity was found for the estimates of SNPs used in MR-IVW and MR-Egger methods (Supplementary Table 7).

**Fig. 5:**
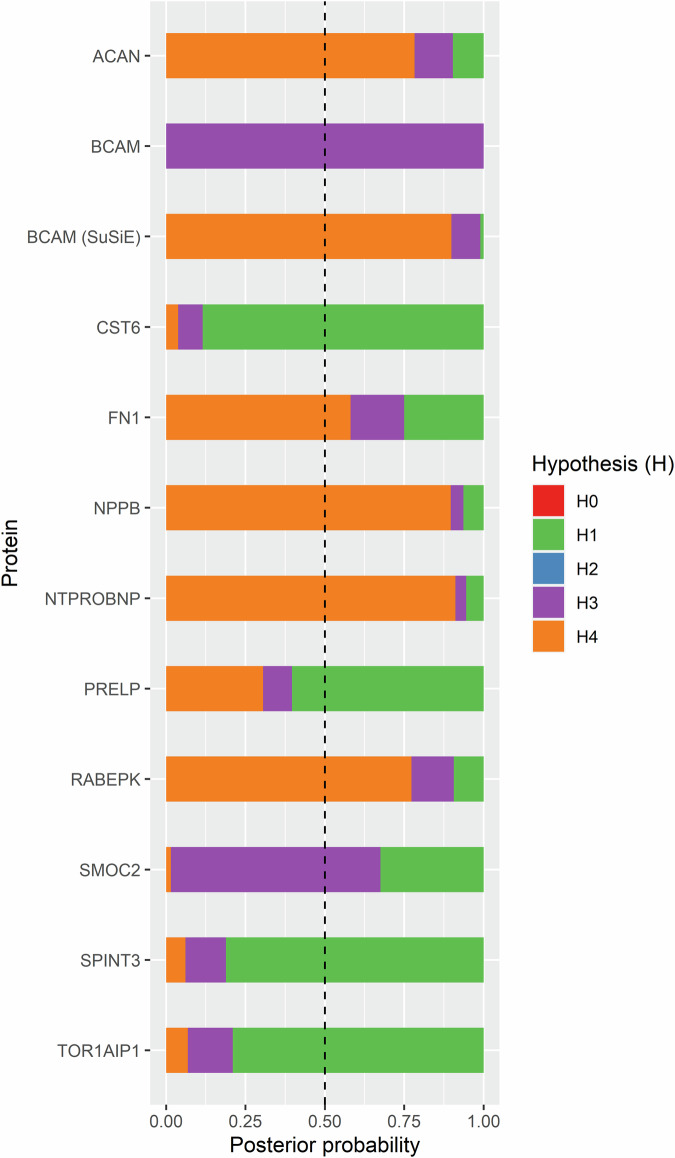
Colocalization analysis of proteins of interest and intima-media thickness (IMT). The figure shows the posterior probability of five exclusive hypotheses to support whether proteins of interest and IMT share the same causal genetic variants in the defined genomic region.

**Fig. 6:**
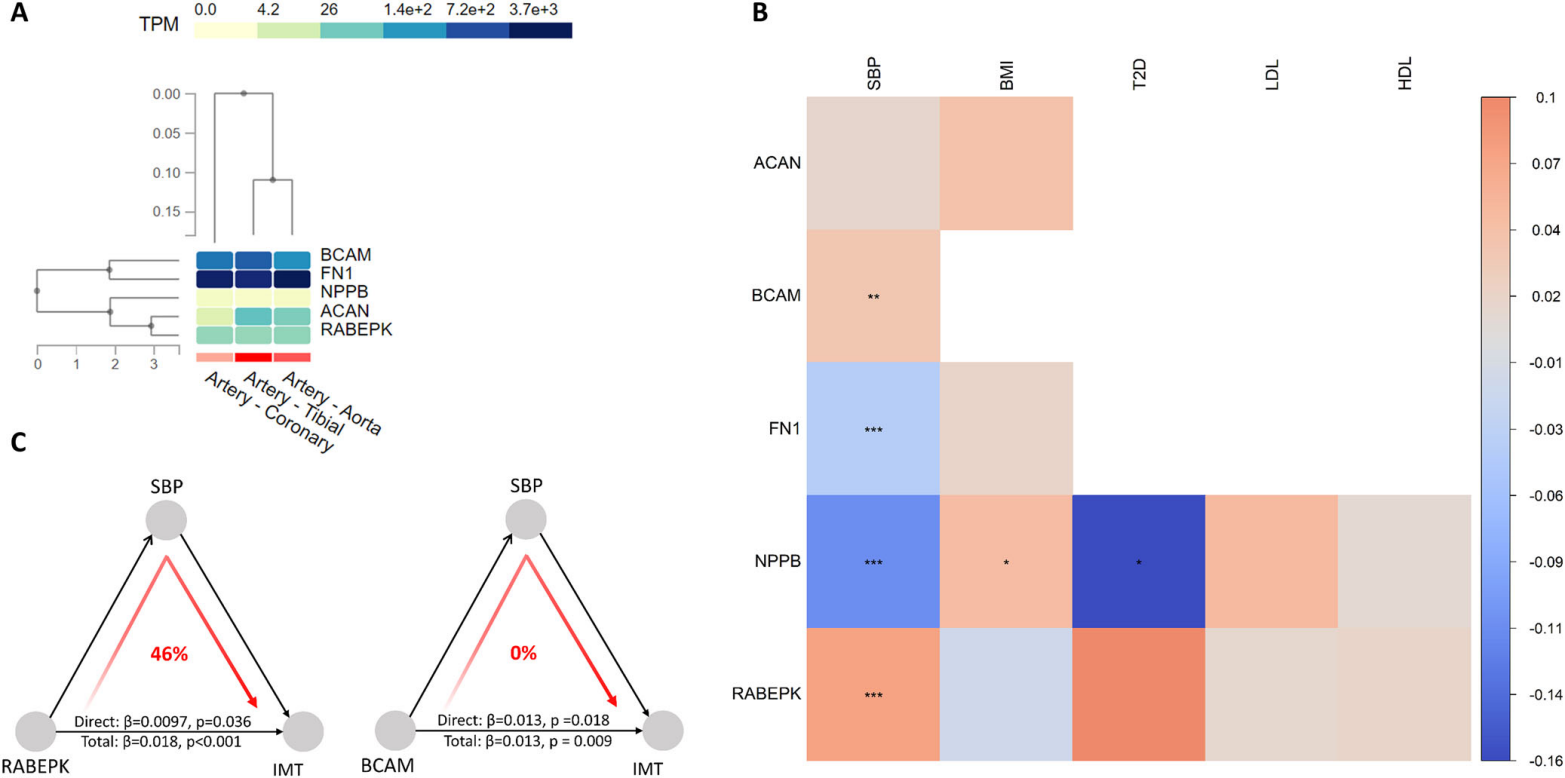
Genetic analyses of proteins of interest. **A** Expression analysis in three relevant arteries for genes of five proteins being significant in the Mendelian randomization (MR) analysis vs the intima-media thickness (IMT). **B** Heat-map of MR-Wald ratio estimate of selected proteins with traditional risk factors of IMT. Significant levels are expressed as ***, **, * or lack of * if nominal *p*-value is <0.001, <0.01, < 0.05 or > 0.05. A blank square indicates that no association was estimated because of lack of genetic instruments. **C** Mediation analysis of the effect of proteins on IMT via SBP using MR.

Of the 5 proteins, NTproBNP/NPPB showed strong evidence of colocalization with IMT (PP.H4 > 80%) as shown in Fig. 5, while RABEPK and ACAN showed medium evidence (PP.H4 > 70%). FN1 showed a PP.H4 of 58% in the colocalization analysis. BCAM showed no evidence of colocalization using the traditional method (Supplementary Table 8), but showed a strong evidence of colocalization (PP.H4 of 90%) when using the SuSiE method (Supplementary Table 9). As could be seen in Fig. 6A, all but NPPB were expressed in arterial tissues.

Of the 63 proteins found significant in the observational analysis, seven showed *p* < 0.05 in the MR analysis (CST6, DSG4, FDX1, PRELP, SMOC2, SPINT3, TOR1AIP1). However, DSG4 and FDX1 showed opposite signs of the estimates in the observational and MR analyses. Five of the seven proteins excluding DSG4 and FDX1 were further submitted for colocalization analysis but none showed evidence (PP.H4 < 0.5) of colocalization (Fig. 5).

Out of the five proteins significantly associated with IMT in the MR analysis, all except ACAN were also associated with SBP (*p* < 0.05), where the associations of RABEPK (beta = 0.07, *p* < 0.001) and NPPB (beta = −0.11, *p* < 0.001) were more pronounced (Fig. 6B, Supplementary Table 10). NPPB was also positively and inversely associated with BMI and T2D risk, respectively.

### The effect of proteins on IMT mediated by SBP using MR analysis

As shown in the Fig. 6C, the MR-IVW estimate of the association of RABEPK with IMT was 0.018 (*p* < 0.001). After adjusting for SBP, the estimate was 0.0097 (*p* = 0.036). Thus, 46% of the effect of RABEPK on IMT was mediated by SBP. However, no effect was mediated via SBP for the association of BCAM with IMT.

### Druggability

For the proteins colocalized with IMT, 3 out of 4 proteins have the druggability tiers. FN1, ACAN and BCAM were listed as Tier 1, Tier 3 A and Tier 3 A, respectively. Only FN1 was targeted by drug in the clinical trial but not for atherosclerosis-related outcomes (Supplementary Table 11).

## Discussion

The present study showed that traditional risk factors for CVD were related to IMT in the expected manner when using measured levels or PRS. Using MR, support for a causal role was only seen for LDL-cholesterol, BMI and SBP. Plasma levels of 63 proteins were related to IMT whereas 17 were replicated in the observational analyses, but using MR and colocalization, support for a causal role was only seen for 5 proteins. However, only the RABEPK- IMT association showed concordant direction in the observational and MR analyses.

Using observational data, it has previously been shown that the traditional risk factors for CVDs, like myocardial infarction and ischemic stroke, also are linked to a thick IMT^7^.

This picture is reproduced in the present study using observational data and PRS, but in the two-sample MR analysis only LDL-cholesterol, BMI and SBP were causally linked to IMT, while HDL-cholesterol and diabetes were not. Thus, using genetic information in two different ways (PRS and MR) gave diverging results for diabetes and HDL-cholesterol.

A causal role for HDL-cholesterol in myocardial infarction has been questioned since early genetic studies did not support a causal role for HDL-cholesterol^11,12^. Also the failure of HDL-rising drugs to improve CVD outcomes has raised concerns of a causal role of HDL-cholesterol in atherosclerotic diseases, as reviewed in^26^. Regarding diabetes, this was a more unexpected finding. Using the same genetic loci as instrumental variables for diabetes, we have previously shown genetically determined diabetes to be linked to both myocardial infarction and stroke, two atherosclerotic diseases, in similar MR studies as used in the present evaluation^16^. Since the occurrence of myocardial infarction and ischemic stroke are most often the result of an atherosclerotic plaque rupture and subsequent clot formation and arterial conclusion, it might be that diabetes is mainly a causal risk factor for these latter events and maybe not so much involved in early vascular pathology.

The discrepant findings for PRS and MR regarding the role of diabetes for IMT also raise questions how these two ways to use genetic information differ. One obvious difference is that the PRS used in the present evaluation is a PRS for type 2 diabetes, while the GWAS for diabetes used in the MR evaluation does include all kinds of diabetes. However, the vast majority of the diabetic cases included in the GWAS for diabetes are type 2, since the majority of subjects were middle-aged or elderly. Another difference is that having a high value at a PRS for diabetes is seen in a number of subjects with normal glucose tolerance, as well as in a number of subjects with overt diabetes at the instant when data are collected, as compared to the MR situation in which the cases in the diabetes GWAS all have overt diabetes.

Of the proteins suitable for MR analysis, only 5 showed evidences of a causal role for IMT when adjustment for multiple testing was performed (BCAM, NT-proBNP/NPPB, RABEPK, ACAN, FN1), but none of those were amongst the 63 proteins identified in the observational analysis. Colocalization analysis supported a causal role for all of those proteins, but *NPPB* was not found to have expression in arteries.

A previous MR analysis also identified NT-proBNP to be inversely related to IMT^25^. In that study, measured NT-proBNP levels were positively related to IMT (in the observational setting) and therefore the relationship between NT-proBNP and IMT was regarded not to be causal. It is more likely that a thick IMT representing a deranged hemodynamic situation that by an increased volume or pressure load on the myocardium results in increased production of NT-proBNP (and BNP (*NPPB*)). It was also seen in the expression analysis in Fig. 5A that NPPB was not abundantly expressed in the evaluated arteries.

Rab9 effector protein with kelch motifs (RABEPK), also called only Rab9, is a protein known to be involved in Retrograde transport at the Trans-Golgi Network and vesicle-mediated transport, as well as autophagosome formation in unconventional macroautophagy, as reviewed by Zhang et al.^27^. Genetic variation in this gene has previously been linked to cigarette use, drug abuse, bipolar disorders, as well as BMI^28^, but its role in atherosclerosis is less known. However, as recently reviewed by Nah^29^, RABEPK could by its role in alternative mitophagy potentially be involved in several myocardial disorders. In UK Biobank, the measured levels of RABEPK were not closely related to any of the traditional CVD risk factors (R^2^ < 1% for all comparisons, results not shown in detail). A look-up in the public databases regarding the potential druggability of RABEPK yielded no findings. Thus, based on the above-mentioned findings, RABEPK might well be a target worthwhile to be explored in future studies and drug development for vascular diseases.

The basal cell adhesion molecule (BCAM), also denoted Lutheran blood group glycoprotein (Lu), is a transmembrane receptor with five immunoglobulin-like domains and belongs to the immunoglobulin gene family. BCAM is involved in blood cell–endothelium interactions, such seen in sickle-cell anemia, polycytemia vera and retinal-vein occlusion, as reviewed by Wautier and Wautier^30^. However, the role of BCAM in CVD in not known. Genetic variation in the BCAM gene has mainly been linked to Alzheimer´s disease^31^ and blood lipid levels^32^. However, measured BCAM levels were not closely related to any of the traditional CVD risk factors (R^2^ < 1% for all comparisons) in UK Biobank. This protein has been judged as a Tier 3 A target of drug.

Aggrecan (ACAN) is a large chondroitin sulfate proteoglycan of the extracellular matrix. This protein forms giant hydrated aggregates with hyaluronan in the extracellular matrix (ECM). ACAN is a major component of cartilage, but is also expressed the cardiovascular system. It is expressed in preatherosclerotic lesions in human carotid arteries, but was reduced in calcified lesions^33^. Aggrecan has in another study been expressed in both stable and unstable advanced plaques^34^. Genetic variation in ACAN has mainly been linked to height^35^, and other anthropometric index, such as waist circumference^36^. In our analysis of measured ACAM levels in UK Biobank, the levels were inversely related to LDL-cholesterol (R^2^ = 2%) and inversely to BMI (R^2^ = 3%). ACAM has been judged as a Tier 3 A target of drug and an experimental compound is available.

Fibronectin 1 (FN1) linking matrices and cells and is found in both cell surface and extracellular matrix in many types of cells. As reviewed by Tang et al, fibronectin is involved in wound healing, hematopoiesis, infections, as well as cancer development^37^. A role of fibronectin 1 has also been suggested for atherosclerosis in which this protein is expressed mainly in the intima and is involved in the transformation of vascular smooth muscle cell to a secretory type of cell^38^. In a cross-sectional human study, low levels of fibronectin 1 was found in patients with coronary heart disease (CHD), but the levels of this protein was not related to the severity of CHD at coronary angiography^39^. SNPs in this gene have been linked to CHD^40^ and a drug, Ocriplasmin, is on the market since a decade for the treatment of symptomatic vitreomacular adhesion. In UK Biobank, measured levels of FN1 is associated with blood pressure (R^2^ = 3%), BMI (R^2^ = 5%) and LDL-cholesterol (R^2^ = 3%).

Since correction for multiple testing might produce false negative results, we evaluated if any of the 63 proteins found related with IMT in the observational analysis showed same directions of the associations in the MR analysis, and also showed *p* < 0.05, even though FDR was >0.05. Five such proteins were found (CST6, PRELP, SMOC2, SPINT3 and TOR1AIP1). To further examine if these proteins could be causally related to IMT, we performed colocalization analysis, but none of those met the colocalization criterion.

It may raise some concerns that the observational and genetic results did not converge as no overlapped proteins were found in both. The reasons behind this are multi-factorial, including heterogeneity of biases in different methods and of statistical estimation approaches. It has been recognized that MR is instinctively less biased than the observational study design, and some of MR results were even comparable to the randomized controlled trial (RCT) studies^41,42^. Our MR findings of NTproBNP/NPPB associated with IMT can serve as a reassurance that our MR results are meaningful to some extent because NTproBNP is a well-established biomarker of CVD used in clinical practice^43^. The MR association of NTproBNP was also supported by the strong colocalization evidence.

In the present study, we used FDR < 0.05 for the age and sex-adjusted model and nominal *p* < 0.05 for the multiple-adjusted model as significance levels in the discovery phase in UK biobank, given that we could validate most of the proteins in the independent POEM sample. It might be argued that we should have applied also FDR < 0.05 for the multiple-adjusted model. If FDR < 0.05 was applied also for the multiple-adjusted model, no protein would be found to be related IMT, which obviously not is the case, given that we have validated 11 (out of 86 tested) proteins to be linked to IMT in our previous study 25.

The strength of the present study is the large number of subjects in whom IMT has been measured together with traditional risk factors and PRS for those risk factors. The number of individuals with parallel protein measurements was smaller, but is still comparable with the largest study conducted so far^25^ and the protein-IMT associations were replicated in an external cohort, although not every protein was measured in the replication cohort. One limitation is that the imaging of IMT was performed some years following the collection of traditional risk factors and proteins, and thus the study design is more longitudinal than cross-sectional in that respect. Changes in risk factors and proteins may well have occurred during this time gap, but any such deviations would possibly only lead to underestimations of the evaluated relationships, and the analysis of the measured traditional risk factors with IMT showed the expected results compared to previous studies in this area. These proteins were also replicated in an independent cohort where IMT, proteins and risk factors were all measured at the baseline. Another limitation is that the discordant results found by observational and genetic analyses may raise concerns over the screening procedure of proteins of interest, and thus interpretation of such results should be cautious. Last, no analysis was conducted for carotid plaque which has been considered a superior measurement to IMT, because of lack of observational data of carotid plaque in the UK Biobank. However, both clinical trials and observational studies have implied strong evidence to support IMT as a useful surrogate marker for cardiovascular risk^44^. Thus our results are of great value to help us further understand vascular biology and atherosclerotic physiopathology.

In conclusion, MR suggested that LDL-cholesterol, BMI, diabetes and SBP are causally related to IMT. MR also identified five proteins of interest for IMT, but further analyses suggested mainly RABEPK to be an interesting target deserving further investigations.

## Methods

### Study samples

The UK Biobank study was approved by the UK North West Multi-Centre Research Ethics Committee (Project Application Number 90143) and the Swedish Ethical Review Authority (Nr. 2023-00148-01), and performed in accordance with the Declaration of Helsinki and other relevant guidelines/regulations. All participants provided written informed consent.

The PEOM study was approved by the Ethics committee of Uppsala University and all participants gave their written informed consent.

UK Biobank is a large, multi-center, prospective cohort study conducted across the UK (https://www.ukbiobank.ac.uk). In 2006–10, over 500,000 individuals aged 40–69 years underwent physical measurements, and blood samples were biobanked for later analysis of genes and biomarkers. The present study used data from the 50,704 individuals with an available measurement of IMT. Participants information on sex, age, and race was self-reported.

Fasting glucose, HbA1c, LDL- and HDL-cholesterol and creatinine were measured by a Beckman coulter AU5800, by standard methods. Blood pressure was measured twice in the sitting position with the automated Omron device. Glomerular filtration rate was estimated (eGFR) by the updated CKD-EPI formula^45^. Body mass index (BMI) was calculated from measured height and weight (weight/height^2). Ethnicity was categorized into four groups; White, Black, Asian, and other. Townsend social deprivation index was used as a marker of socioeconomic status. Smoking was categorized as never, previous or current. PRSs for 28 diseases and 25 quantitative traits have previously been calculated on the individuals in UK Biobank by Thompson and co-workers^46^. These data are, as other variables, available for scientists upon request from UK Biobank. The Standard PRS set of 28 diseases and 8 quantitative traits was generated from external GWAS data. The current study used the “Standard PRSs” calculated in the majority of subjects. PRS algorithms were built from trait-specific meta-analyses using a Bayesian approach, where appropriate combining data across multiple ancestries and related traits. Per-individual PRS values were calculated as the genome-wide sum of the per-variant posterior effect size multiplied by allele dosage. Further details on the calculations of the PRSs could be found in the reference^46^. The present study used the PRS for diabetes type2, hypertension, LDL and HDL cholesterol and BMI. Plasma levels of 2923 proteins measured by the Olink proteomics assay Explore (OLINK, Uppsala, Sweden) were determined by the proximity extension assay (PEA) technique in 52,164 UKB participants. An extensive quality control was performed, given in detail in the reference^47^. The far wall of the common carotid artery was scanned by ultrasound (CardioHealth Station, Panasonic Healthcare Corporation of North America, Newark, NJ, USA) at two prespecified angles for each of the two arteries (right 150°, right 120°, left 210°, and left 240°). The mean, min and max of the four measured angles were recorded. The present study used the mean value of the four measurements of maximal IMT. Details on the protocol and quality assurance have previously been published^48^. The measurements of IMT took place on average after 9.6 years following the baseline examination. The overlap between the individuals with protein measurements and the individuals with IMT measurements were 6328 individuals.

The Prospective investigation of ObEsity and Metabolism (POEM) study is a population-based cohort conducted between 2010 and 2016 in Uppsala, Sweden. The cohort is composed of 502 individuals all aged 50 years (50% women). Detailed description has been given in reference^49^. Participants information on sex, age, and race was self-reported. Information on gender and socioeconomic status was not collected. Plasma levels of 1463 proteins measured by the Olink proteomics assay Explore (OLINK, Uppsala, Sweden) were determined by the same PEA technique as in UK Biobank. Proteins detected in less than 75% of the individuals were not used in the analysis. The carotid artery was assessed by external B-mode ultrasound imaging (Acuson XP128 with a 10 MHz linear transducer, Mountain View, California, USA). IMT was evaluated in the far wall in the common carotid artery 1–2 cm proximal to the bulb. The images were digitized and imported into the AMS (Artery Measurement Software, Gothenburg, Sweden) automated software for dedicated analysis of IMT. A 10 mm segment with good image quality was chosen for IMT-analysis from the carotid artery. The value obtained was the mean of around 100 discrete measurements over the 10 mm segment. The given value for carotid artery IMT was the mean value from both sides^50^.

### Selection of genetic instruments

Summary data of each risk factor were obtained from already published GWAS studies: For LDL and HDL, data from the Global Lipids Consortium were used^51^. Data from the GIANT consortium were used for BMI^52^. Data from the DIAGRAM consortium were used for diabetes^53^. For systolic blood pressure (SBP), own calculations using UK biobank were used. Only independent (in low linkage disequilibrium, R^2^ < 0.01) SNPs with *p* < 5e-8 were used as instrument.

We obtained the summary-level GWAS statistics from the UKB-Pharma Proteomics Project (PPP) which comprehensively mapped protein quantitative trait locus (pQTL) of 2923 proteins in 54,219 UK Biobank participants^47^. We only used the cis-pQTL SNPs for our MR analysis of proteins with various outcomes to minimize the likelihood of horizontal pleiotropy. The genetic instruments were curated from three sources: 1). The cis-pQTLs defined in the combined cohort; 2). If the sentinel SNP (cis-pQTL) is multiallelic in the combined cohort, the meta-analyzed statistics of the cis-pQTL SNP in the discovery and replication cohorts were used; 3). For a cis-pQTL SNP being multiallelic in both combined and discovery-replication cohorts, the SNP at the cis-region (defined as the 1 Mb pair window of the protein-coding gene) with the lowest *p*-value and being biallelic in the combined cohort was used as the instrument. Detailed definition of the combined and the discovery-replication cohorts can be found in the original publication^47^. Totally, 1676 cis-pQTL SNPs were included as genetic instruments. The minimum value of F-statistic for all instruments was 41.

### Data source of outcome GWAS

Summary-level GWAS statistics of the ultrasound-measured carotid artery IMT (*n* = 71,128) were obtained from a GWAS of Europeans^6^.

### Statistics

#### Observational analyses

The measured risk factors were transformed to the standard deviation (SD)-scale in order to be comparable with the PRS. Also IMT was transformed to the SD scale. Linear regression models between IMT and either measured levels of the risk factors (history of diabetes, systolic blood pressure, LDL and HDL cholesterol and BMI) or PRS for the risk factors (diabetes type 2, hypertension, LDL and HDL cholesterol and BMI) were applied. Included in these multiple models were also the covariates age, sex, smoking, Townsend deprivation index and race. *P* < 0.05 was regarded as significant in these multiple models.

Linear regression models between IMT and each of the 2923 available proteins (one by one) were applied in two sets of adjustment, including model 1 for age, sex and fasting time and model 2 for age, sex and fasting time plus smoking, Townsend deprivation index and race and the CVD risk factors diabetes, systolic blood pressure, LDL and HDL cholesterol, BMI, and eGFR. In order to be deemed significant, the estimate of proteins should show a false discovery rate (FDR) < 0.05 in model 1 and *p* < 0.05 in model 2. Measured levels of each of proteins of interest were inverse normalized rank transformed and were then associated with IMT in the POEM sample using linear regression models, including sex as confounder (age same in all subjects). *P* < 0.05 was considered significant in this validation analysis.

#### Genetics

Multivariable MR was performed with IMT as the outcome and diabetes, systolic blood pressure, LDL and HDL cholesterol and BMI as the exposures. The inverse-variance weighted (IVW) method was considered as the primary analysis, and MR Egger as a sensitivity test. *P* < 0.05 was considered to be significant for IVW, and we also demanded that the estimate of the MR Egger test to be in the same direction as the estimate of IVW. STATA 16.1 was used for the calculations of this part of the MR studies. We used a linear fixed-effects model (R package “metafor”)^54^ to generated the meta-analyzed statistics of the cis-pQTLs found in the discovery and replication cohorts where the weighted estimation with IVW was implemented. Since we only included the sentinel cis-pQTL SNP per protein, only the Wald-ratio method was used for the MR analysis. Multiple testing was addressed by the FDR controlling procedure and adjusted *p*-value (shortly as FDR) < 0.05 was regarded statistically significant. To examine if the hypothetic causal direction was oriented from proteins to IMT, the MR-Steiger test was used. For the protein-IMT associations found significant by MR, further sensitivity analyses employing multiple GWAS-significant (*p*-value < 5e-8) independent (in low linkage disequilibrium, R^2^ < 0.01) SNPs with F-statistic > 10 at the cis-locus (defined as the above) were performed including MR-IVW, MR-Egger, MR-Weighted median and MR-Weighted mode methods. Horizontal pleiotropy was examined based on the *p*-value of the MR-Egger intercept. Heterogeneity of the instruments was assessed by Cochrane’ Q value. Nominal *p*-value < 0.05 was regarded significant in the sensitivity analyses. All the MR analyses were conducted by using R package “TwoSampleMR”^55^.

To examine whether the significant associations of proteins with IMT found by MR were confounded by linkage disequilibrium, we furthered applied genetic colocalization for proteins of interest (FDR < 0.05) and IMT. We included the SNPs at the cis-region (as above) of each protein and defined a posterior probability (PP) of the hypothesis that two traits of interest are colocalized (H4) > 0.8 as strong support of colocalization whereas 0.5 < PP.H4 ≤ 0.8 as medium support. Three prior probabilities were defined as follows: p1 (a SNP is associated with trait 1) = 1e-4; p2 (a SNP is associated with trait 2) = 1e-4; For p12 (a SNP is associated with both traits), a more conservative value 5e-6 was used for improved robustness^56^. To relax the assumption by the traditional *coloc* method that at most one causal variant per trait, we further performed colocalization analysis in the framework of Sum of Single Effects (SuSiE) where applicable^57^. The sensitivity of the inference to variation of p12 was examined by visualization. The colocalization analyses were conducted by using R package “coloc”^58^.

We used the same genetic instruments of the proteins in the last step and evaluated the association of genetically predicted levels of proteins and risk factors of IMT by the MR-Wald ratio method and *p*-value < 0.05 was deemed significant.

To disentangle the mediate effect of proteins (RABEPK and BCAM) on IMT through SBP, we first employed multiple cis-SNPs of each protein (the same source as used for the MR sensitivity analyses of protein and IMT) as instruments to estimate the effect of proteins on IMT by univariable MR-IVW method (β1), which was regarded as *the total effect*. In the second step, using the same source of instruments as in the first step, the multivariable MR method was implemented to estimate *the direct effect* of proteins on IMT (β2), adjusted for SBP. The effect mediated by SBP was thus (β1- β2)/ β1. The *mvmr* function in the R package “TwoSampleMR” was used for the multivariable MR analysis.

#### Lookups of Genotype-Tissue Expression (GTEx) and druggability

For proteins of interest, we further looked up the gene expression levels in the human artery tissue at the GTEx Portal (https://gtexportal.org/home, dbGaP accession number phs000424.v8.p2). The tier of the proteins’ druggability was assigned according to the previous work by Finan et al.^59^ where Tier 1 as proteins targeted by approved drugs and drugs in clinical development; Tier 2 as proteins closely related to drug targets or with associated drug-like compounds; Tier 3 as extracellular proteins and members of key drug target families. Detailed drug development information was curated from DrugBank (https://go.drugbank.com/), https://clinicaltrials.gov/ and ChEMBL (https://www.ebi.ac.uk/chembl/) if applicable.

## Supplementary information

Supplementary file

Supplementary tables

## Data Availability

I. Observational data were obtained from the UK Biobank. The UK Biobank and its data is an open research resourse available following submission of a research plan at https://www.ukbiobank.ac.uk. According to the Swedish law, individual health data cannot be made publicly available (POEM). Data supporting the findings of the present study are available for researchers at a reasonable request to L.L. (email: lars.lind@medsci.uu.se). II. Summary-level GWAS data of • proteins available at https://metabolomips.org/ukbbpgwas/. • IMT available at https://www.ncbi.nlm.nih.gov/projects/gap/cgi-bin/study.cgi?study_id=phs000930.v6.p1.

## Code availability

No customized code generated for analysis in this work. STATA 16.1 was used for the calculations based on the observational data. R (version 4.1.0) was used for the MR and colocalization analyses.
