# Supplementary material for: Associations between carotid artery intima-media thickness, traditional risk factors and proteins
Source: NPJ Cardiovasc Health. 2025 Jul 2;2:35. doi: 10.1038/s44325-025-00073-7 (PMC12912406; doi:10.1038/s44325-025-00073-7)
Supplement: Supplementary file 1 — Supplementary file [file 44325_2025_73_MOESM1_ESM.pdf]

Supplementary table legends

Supplementary Table 1. The information for genetic instruments used for Mendelian randomization (MR) analysis.

Supplementary Table 2. Relationships between intima-media thickness (IMT) of the carotid artery and measured levels (observational), or genetically predicted levels of the traditional risk factors.

Supplementary Table 3. The association of measured levels of proteins and IMT.

Supplementary Table 4. Pathway enrichment analysis of 63 proteins significantly associated with IMT in the observational analysis.

Supplementary Table 5. The validated associations of proteins of interest with IMT in the POEM study.

Supplementary Table 6. The associations of genetically predicted levels of proteins with IMT by MR Wald ratio method.

Supplementary Table 7. The senstivitiy analyses of the significant associations (p-value corrected by FDR < 0.05) of proteins and IMT found by the MR-Wald ratio method.

Supplementary Table 8. Colocalization analysis of proteins and IMT by the traditional method.

Supplementary Table 9. Colocalization analysis of BCAM and IMT by the SuSiE method.

Supplementary Table 10. The associations of genetically predicted levels of proteins with risk factors of IMT by the MR-Wald ratio method.

Supplementary Table 11. The druggability of proteins of interest.
